# Demethylzeylasteral (T-96) initiates extrinsic apoptosis against prostate cancer cells by inducing ROS-mediated ER stress and suppressing autophagic flux

**DOI:** 10.1186/s40659-021-00350-6

**Published:** 2021-09-06

**Authors:** Dong-lin Yang, Ya-jun Zhang, Liu-jun He, Chun-sheng Hu, Li-xia Gao, Jiu-hong Huang, Yan Tang, Jie Luo, Dian-yong Tang, Zhong-zhu Chen

**Affiliations:** 1 College of Pharmacy, National & Local Joint Engineering Research Center of Targeted and Innovative Therapeutics, Chongqing Key Laboratory of Kinase Modulators as Innovative Medicine, Chongqing University of Arts and Sciences, Chongqing, 402160 China; 2 College of Pharmaceutical Sciences and Chinese Medicine, Southwest University, Chongqing, 400715 China

**Keywords:** T-96, CaP, ER stress, Apoptosis, Autophagic flux, Cisplatin

## Abstract

**Background:**

Demethylzeylasteral (T-96) is a pharmacologically active triterpenoid monomer extracted from *Tripterygium wilfordii* Hook F (TWHF) that has been reported to exhibit anti-neoplastic effects against several types of cancer cells. However, the potential anti-tumour effects of T-96 against human Prostate cancer (CaP) cells and the possible underlying mechanisms have not been well studied.

**Results:**

In the current study, T-96 exerted significant cytotoxicity to CaP cells in vitro and induced cell cycle arrest at S-phase in a dose-dependent manner. Mechanistically, T-96 promoted the initiation of autophagy but inhibited autophagic flux by inducing ROS-mediated endoplasmic reticulum (ER) stress which subsequently activated the extrinsic apoptosis pathway in CaP cells. These findings implied that T-96-induced ER stress activated the caspase-dependent apoptosis pathway to inhibit proliferation of CaP cells. Moreover, we observed that T-96 enhances the sensitivity of CaP cells to the chemotherapeutic drug, cisplatin.

**Conclusions:**

Taken together, our data demonstrated that T-96 is a novel modulator of ER stress and autophagy, and has potential therapeutic applications against CaP in the clinic.

**Supplementary Information:**

The online version contains supplementary material available at 10.1186/s40659-021-00350-6.

## Background

Prostate cancer (CaP) is one of the cancers associated with high incidence and mortality rates among men worldwide [1]. Increasing evidence from studies show that the development of CaP is associated with perturbations in the androgen receptor (AR) signalling pathway [2, 3]. AR is a ligand-dependent transcription factor belonging to the nuclear receptor family. Currently, androgen deprivation is the main treatment strategy for early stage of CaP since initial CaP cell proliferation is dependent on androgens. However, there is no effective therapy against castration-resistant CaP, a lethal form of CaP that is androgen-independenct [4]. Despite the great advances in the therapeutic strategies against CaP, there are increased incidences of recurrence and metastasis of CaP [5]. Thus, there is need to identify novel agents that are effective in suppressing the development and progression of CaP with minimal side effects, to improve survival rates among CaP patients.

The endoplasmic reticulum (ER) is the largest intracellular organelle and it plays a vital role in the synthesis, modification, assembly, folding and structural maturation of proteins [6]. When the ER protein-folding capability is exceeded, unfolded or misfolded proteins accumulate in the ER lumen and can lead to the occurrence of ER stress [7, 8]. Cells have evolved a highly protective signal transduction pathway, referred to as unfolded protein response (UPR), to alleviate ER stress by enhancing protein folding ability, reducing protein transduction rate and degrading unfolded and misfolded proteins [9, 10]. ER stress can promote UPR-induced autophagy in damaged cells, which causes autophagic vesicles to engulf the impaired ER. The UPR signal is mainly initiated through the activation of three transmembrane proteins located on ER: inositol-requiring enzyme 1α (IRE1α), double-stranded RNA-dependent protein kinase (PKR)-like endoplasmic reticulum kinase (PERK) and activating transcription factor 6 (ATF6) [11, 12]. Initiation of the UPR pathway is a mechanism by the stressed cells to accommodate and survive the ER stress through transcriptional and translational reprogramming [13]. However, if protein folding homeostasis cannot be restored in the ER, UPR initiates an alternative signalling pathway, termed terminal UPR, which eventually promotes apoptosis induced by the toxicity of unfolded/misfolded proteins [9, 10, 14]. Therefore, the induction of ER stress above a critical threshold by novel compounds and the subsequent activation of the programmed cell death signalling pathway may be an effective strategy for cancer therapy.

*Tripterygium wilfordii* Hook F (TWHF), commonly known as “lei gong teng” or “thunder god vine”, has been reported to treat a wide range of autoimmune and inflammatory diseases such as rheumatoid arthritis, ankylosing spondylitis, systemic lupus erythematosus and psoriasis [15–18]. The major effector components of TWHF are triptolide and celastrol, which are diterpenoid and triterpenoid compounds, respectively. Triptolide and celastrol have been shown to have anti-tumour activities by inducing apoptotic or autophagic cell death as well as cell cycle arrest in a series of cancer cells [17–22]. Demethylzeylasteral (T-96), a triterpenoid monomer compound, is a component of TWHF that is associated with considerably lower toxicity effects compared to triptonide, celastrol and triptolide in *in vitro* and in vivo studies [23]. Previous studies have indicated that T-96 is capable of suppressing cell proliferation, metastasis and angiogenesis, as well as promoting cell apoptosis in various malignant carcinomas, such as melanoma [24], glioblastoma [25] and breast cancer [26]. Interestingly, T-96 is also involved in the inhibition of pancreatic cancer proliferation through autophagy-induced apoptosis, and significantly increases chemosensitivity to gemcitabine [27]. However, there have been no reports on the regulatory mechanisms underlying the anti-tumour effects of T-96 in human CaP.

In the present study, we evaluated the anti-tumour effects of T-96 against CaP and the underlying mechanisms involved in the inhibition of CaP cell growth. We further investigated whether T-96 increases the chemo-sensitivity of CaP cells to cisplatin. Our results demonstrated for the first time that T-96 inhibited cell proliferation through cell cycle arrest, initiating autophagy and inducing ER stress-mediated apoptotic cell death.

## Materials and methods

### Reagents and antibodies

Demethylzeylasteral (T-96, molecular formula: C29H36O6, molecular weight: 480, T3418), Z-VAD-FMK (T6013), Rapamycin (T1537), Chloroquine (T8689) and Cisplatin (T1564) were purchased from TOPSCIENCE (Shanghai, China). Penicillin–Streptomycin, 3-(4,5-Dimethylthiazol-2-yl)-2,5-diphenyltetrazolium bromide (MTT), and propidium iodide (PI) were purchased from Sigma-Aldrich (MO, USA). Cell culture medium including DMEM (SH30022.01B) and F12K (SH30526.01) were sourced from HyClone (Logan, USA) while fetal bovine serum (FBS) was purchased from Natocor (Cordoba, Argentina). All the primary antibodies were bought from Cell Signaling Technology (MA, USA), while the secondary antibodies were purchased from LI-COR Biosciences (NE, USA).

### Cell culture

Human CaP cell lines DU145 and PC3, and normal adult prostatic epithelial cell line PNT1A were obtained from the American Type Culture Collection (ATCC, VA, USA). All cell lines were mycoplasma-free and were authenticated using STR profiling. DU145, PC3 and PNT1A cells were cultured in Dulbecco’s modified Eagle’s medium (DMEM)/High glucose culture medium, Kaighn’s modification of Ham’s F-12 medium and Roswell Park Memorial Institute (RPMI) 1640 medium, respectively, supplemented with 10% fetal bovine serum, 1% Penicillin–Streptomycin at 37 °C in a humidified incubator containing 5% CO_2_.

### Cell viability assay

DU145, PC3 and PNT1A cells were plated into 96-well plates (3000 cells/well) and incubated at 37 °C with various concentrations of T-96, cisplatin, and T96 combined with cisplatin for designated durations. Thereafter, 1% MTT was added (20 µL/well) and incubated at 37 °C for 4 h followed by dissolution of the crystals using DMSO. Absorbance was then detected at 570 nm using a microplate reader (Bio-Tek, VT, USA), and analyzed using GraphPad Prism 7.0. All experiments were performed in triplicates.

### Colony formation assay

DU145 and PC3 cells were plated into six-well plates (500 cells/well) and cultured for 7 days under different treatment conditions. The colonies formed were washed twice with Phosphate Buffered Saline, fixed with 4% Paraformaldehyde for 15 min at room temperature and then stained with 1% crystal violet for 30 min. All statistical measurements were performed in triplicates.

### 5-Ethynyl-20-deoxyuridine (EdU) incorporation assay

The proliferation rate of human CaP cells was investigated using BeyoClick™ EdU Cell Proliferation Kit with Alexa Fluor 555 (C0075S, Beyotime, Shanghai, China) according to the manufacturer’s protocols. Briefly, DU145 and PC3 cells at the logarithmic growth stage were seeded into 24-well plates prior to treatment with different concentrations of T-96 for 48 h. Thereafter, the cells were incubated with 10 µM EdU working solution in the dark room for 2 h at 37 °C. The labeled cells were then fixed with 4% Paraformaldehyde for 20 min, permeabilized with 0.1% Triton-X100 for 10 min, and then stained with click reaction solution in the dark for 30 min. The cells were also incubated with 1 mg/mL 2-(4-amidinophenyl)-6-indolecarbamidine dihydrochloride for 30 min. The images were captured using a fluorescence microscope (Olympus, Tokyo, Japan) and the percentage of EdU-positive cells was calculated using GraphPad Prism 7.0.

### Flow cytometry analysis

The flow cytometry analysis was performed as previously described [28]. Briefly, PI/RNase staining buffer [Becton Dickinson (BD)] was used to assess the cell cycle progression, while AnnexinV-fluoresceine isothiocyanate/PI apoptosis assay kit (C1062S, Beyotime, China) was used to assess apoptosis, according to the manufacturer’s guidelines. Finally, the stained cells were analyzed using BD AccuriTM C6 flow cytometry (BD Biosciences, USA) and FlowJo 7.6 software.

### Western blot analysis

The western blot assay was conducted as previously reported [29]. In brief, whole-cell lysates were obtained by suspending cells in RIPA buffer (P0013B, Beyotime, Shanghai, China), followed by protein quantification using a BCA (Bicinchoninic Acid) Protein Assay Kit (P0010S, Beyotime, Shanghai, China). Immunoreactivity was visualized by an odyssey two-color infrared fluorescence imaging system (LI-COR Biosciences, NE, USA). β-Tubulin was used as the loading control.

### Determination of reactive oxygen species (ROS) formation

Intracellular ROS levels were measured using a ROS Assay Kit (S0033S, Beyotime, Shanghai, China) according to the manufacturer’s protocols. CaP cells treated with T-96 or the control were incubated with serum-free DMEM or F12K containing 2,7-dichlorodi-hydrofluorescein diacetate (DCFH-DA, 10 µM) for 20 min. Subsequently, cell suspensions were centrifuged and washed three times with serum-free DMEM, and then visualized using a fluorescence microscope (IX53/DP80, Olympus Corporation, Japan).

### Ca^2+^ signals measurement

Intracellular Ca^2+^ signaling was determined using Fluo-4 AM (S1060, Beyotime, Shanghai, China) according to the manufacturers’ protocols. CaP cells were collected and washed three times with PBS, and then incubated with 1 µM Fluo-4 AM in PBS for 30 min at 37 °C. Cells loaded with Fluo-4 AM were then washed with PBS and incubated for an additional 20 min to ensure that Fluo-4 AM was completely transformed into Fluo-4. Intensity of fluorescence was captured using a fluorescence microscope (IX53/DP80, Olympus Corporation, Japan).

### Autophagy analysis

Autophagy analysis was performed as described previously [30]. In brief, the lentivirus packaging vectors (Pspax2, pMD2G) and GFP-LC3B/mCherry-GFP-LC3B were co-transfected into HEK293T cells using the Lipo8000 transfection reagent (C0533, Beyotime, Shanghai, China). Viral particles were collected 48 h after transfection and then used to infect CaP cells using Polybrene (10 µg/mL). Subsequently, cells were screened using puromycin (10 µg/mL) to obtain cells with stable expression of GFP-LC3B or mcherry-EGFP-LC3B. The transgenic cells were then treated using different concentrations of T-96, and analyzed using the high content analysis system-operetta CLS™ (PerkinElmer, Waltham, MA, USA).

### Statistical analysis

All experiments were performed in triplicates. GraphPad Prism version 7.0 (GraphPad Software, San Diego, CA, USA) was used for statistical analysis. Data were presented as mean ± Standard deviation, and the ANOVA method was used to compare differences between groups. P < 0.05 was considered to be significant.

## Results

### T-96 exhibits cytotoxic effects against CaP cells

**Fig. 1:**
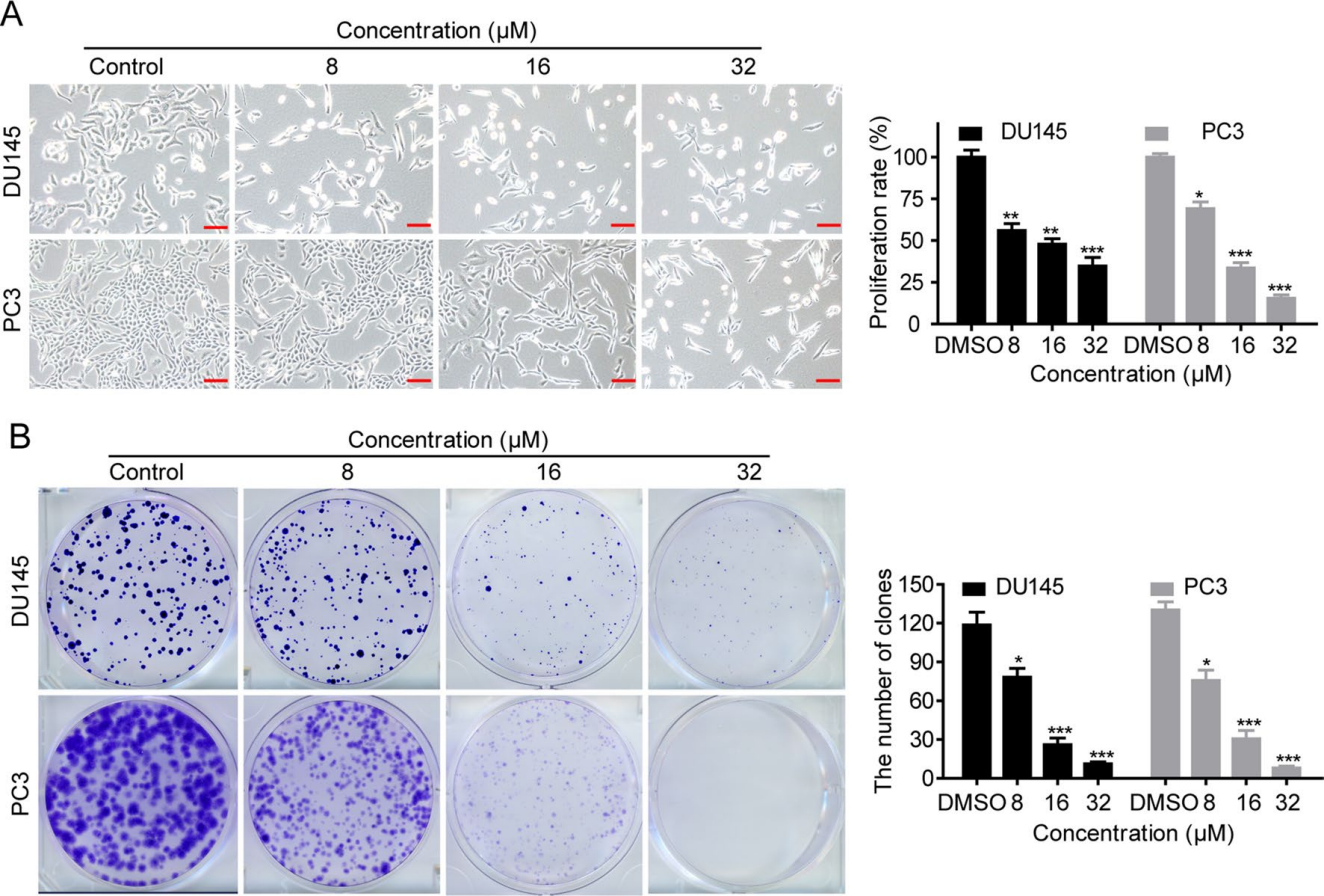
The anti-proliferative effect of T-96 in human CaP lines. **A** Cell morphology of DU145 and PC3 cells after treatment with vehicle (0.05% DMSO) or the indicated concentrations of T-96 for 48 h as captured with a microscope. Scale bar, 100 μm. The histogram shows quantification of the cell proliferation rate. **B** Colony formation assay was used to evaluate the in vitro growth of DU145 and PC3 cells after exposure to the indicated doses of T-96 for 7 days. The colonies were captured as images and the numbers presented using a histogram. All data are presented as the mean ± SD of three independent experiments. *P < 0.05, **P < 0.01 and ***P < 0.001 versus vehicle

**Fig. 2:**
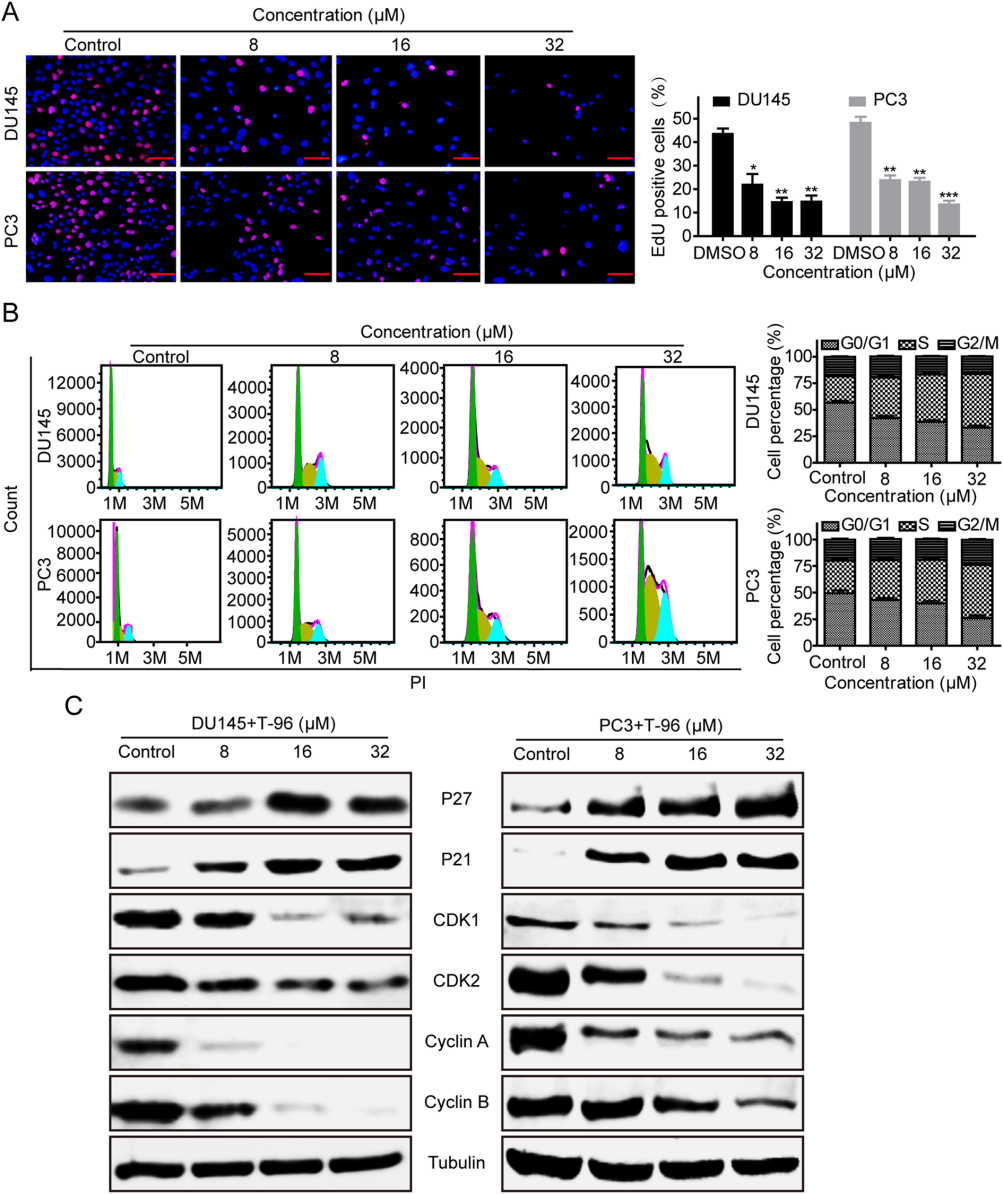
T-96 exhibited anticancer effects by inducing cell cycle arrest at the S phase. **A** T-96 suppressed the proliferation of CaP cells. EdU-staining assay was conducted to access the effect of T-96 treatment for 48 h on cell proliferation. Scale bar, 100 μm. **B** Flow cytometry was used to quantify the proportion of DU145 and PC3 cells in the different stages of the cell cycle in the presence of vehicle or T-96. The percentages of cell population in different conditions were quantified using three independent experiments. **C** Effects of T-96 on the expression levels of S-phase related proteins in DU145 and PC3 cells were examined using western blot after exposure to T-96 for 48 h. β-Tubulin was used as a loading control. All data are presented as the mean ± SD of three independent experiments. “*” represents P < 0.05; “**” represents P < 0.01; “***” represents P < 0.001

The chemical structure of T-96 is shown in Additional file 1: Figure S1A. Since T-96 suppresses the growth of various cancer cells, we investigated if it has anti-cancer effects against CaP cell lines such as DU145 and PC3. We found that T-96 significantly inhibits cell proliferation in a dose- and time-dependent manner (Additional file 1: Figure S1B). The half maximal inhibitory concentration (IC_50_) values of T-96 in DU145 and PC3 were 11.47 µM and 13.10 µM, respectively (Additional file 1: Figure S1C). In contrast, exposure to T-96 was associated with minimal cytotoxicity in normal adult prostatic epithelial cell line PNT1A (Additional file 1: Figure S1D). Cisplatin, an antineoplastic chemotherapeutic agent, was used as a positive control to treat CaP cells with the IC_50_ values in DU145 and PC3 being 6.95 µM and 13.03 µM, respectively (Additional file 1: Figure S1E). Additionally, we found that DU145 and PC3 cell numbers were negatively correlated with the concentration of T-96 used (Fig. 1A). We further conducted colony formation and 5-ethynyl-2′-deoxyuridine (EdU) assays to evaluate the inhibitory effect of T-96 on cell growth and proliferation of CaP cells. As shown in Fig. 1B, exposure to T-96 reduced the size and number of CaP cell colonies in a dose-dependent manner. Similarly, results of EdU-staining indicated that exposure to T-96 significantly deceased DNA synthesis in a dose-dependent manner, implying that T-96 inhibits the growth and survival of CaP cells (Fig. 2A).

### T-96 inhibited cell cycle progression by arresting CaP cells in the S-phase

Cell cycle analysis was conducted to determine the mechanism underlying the anti-proliferative effects of T-96 in CaP cells. Results of flow cytometry, showed that T-96 significantly induced cell cycle arrest at S-phase by decreasing the distribution of DU145 and PC3 cells in G0/G1 phase (Fig. 2B). Since T-96 had induced cell cycle arrest of DU145 and PC3 cells in the S phase, we analyzed its effect on the expression levels of S-phase related proteins to determine if T-96 induced cell cycle arrest through modulation of protein expression. Results of western blot analysis showed that T-96 significantly decreased the levels of Cyclin A, Cyclin B, CDK1 and CDK2, but significantly increased the levels of P21 and P27 in a dose dependent manner in DU145 and PC3 cell lines (Fig. 2C). Taken together, these data demonstrated that T-96 inhibited cell proliferation by inducing cell cycle arrest of CaP cells in the S-phase.

### T-96 activated the extrinsic -apoptosis signalling pathway in CaP cells

**Fig. 3:**
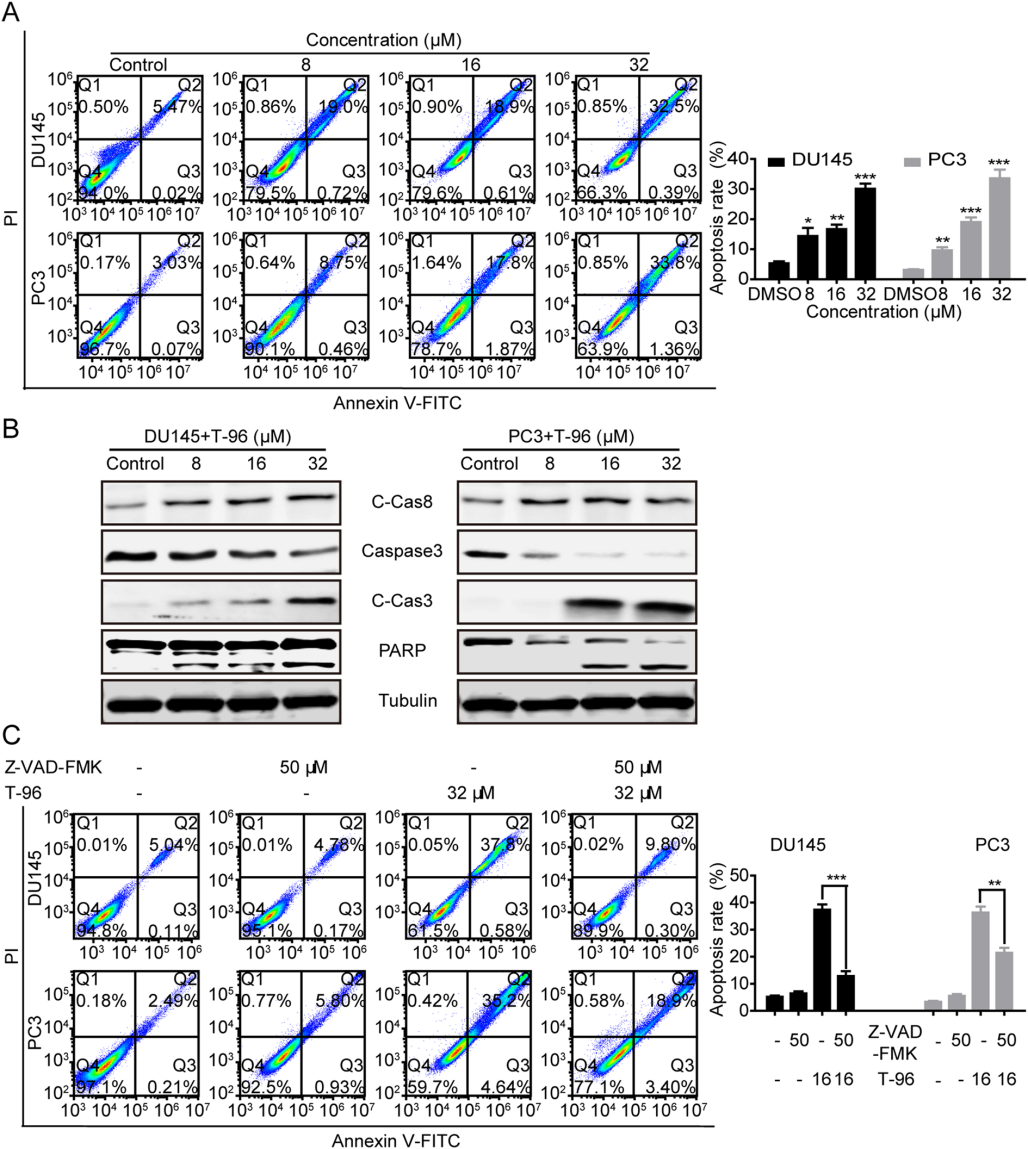
T-96 induced caspase-3-dependent apoptotic cell death in human CaP cells. **A**,** C** Apoptotic cell death in DU145 and PC3 cells was assessed using flow cytometry after treatment with T-96, Z-VAD-FMK (50 µM), or T-96 in combination with Z-VAD-FMK (50 µM) for 48 h. **B** The expression of indicated proteins in DU145 and PC3 cells were examined using western blotting after exposure to T-96 for 48 h. β-Tubulin was used as the loading control. Data represents the mean ± SD of three independent experiments. *P < 0.05, **P < 0.01 and ***P < 0.001

To further determine if T-96 suppressed CaP cell proliferation by activating apoptosis, we conducted Annexin V-FITC/PI assay using flow cytometry after exposing the cells to T-96 for 48 h. As indicated in Fig. 3A, T-96 significantly induced cell apoptosis in CaP cells and increased the proportion of cells in late-phase apoptosis (from 5.47 to 32.5% for DU145 cells, P < 0.001; from 3.03 to 33.8% for PC3 cells, P < 0.001) in a dose-dependent manner. We also assayed the expression levels of proteins associated with the extrinsic-apoptosis signaling pathway to determine if T-96 treatment activates this pathway. T-96 treatment induced a significant increase in the levels of Cleaved Caspase8 (C-Cas8), Cleaved Caspase3 (C-Cas3) and Cleaved PARP in a dose-dependent manner (Fig. 3B). Moreover, a specific caspase3 inhibitor, Z-VAD-FMK, significantly decreased the rate of apoptosis in DU145 and PC3 cells, suggesting that Z-VAD-FMK partially rescues cells from T-96-induced cell apoptosis (Fig. 3C). In summary, these data indicated that T-96 induced apoptosis in CaP cells through the activation of the extrinsic-apoptosis signaling pathway.

### T-96 induced ER stress by promoting the generation of ROS in prostate cells

**Fig. 4:**
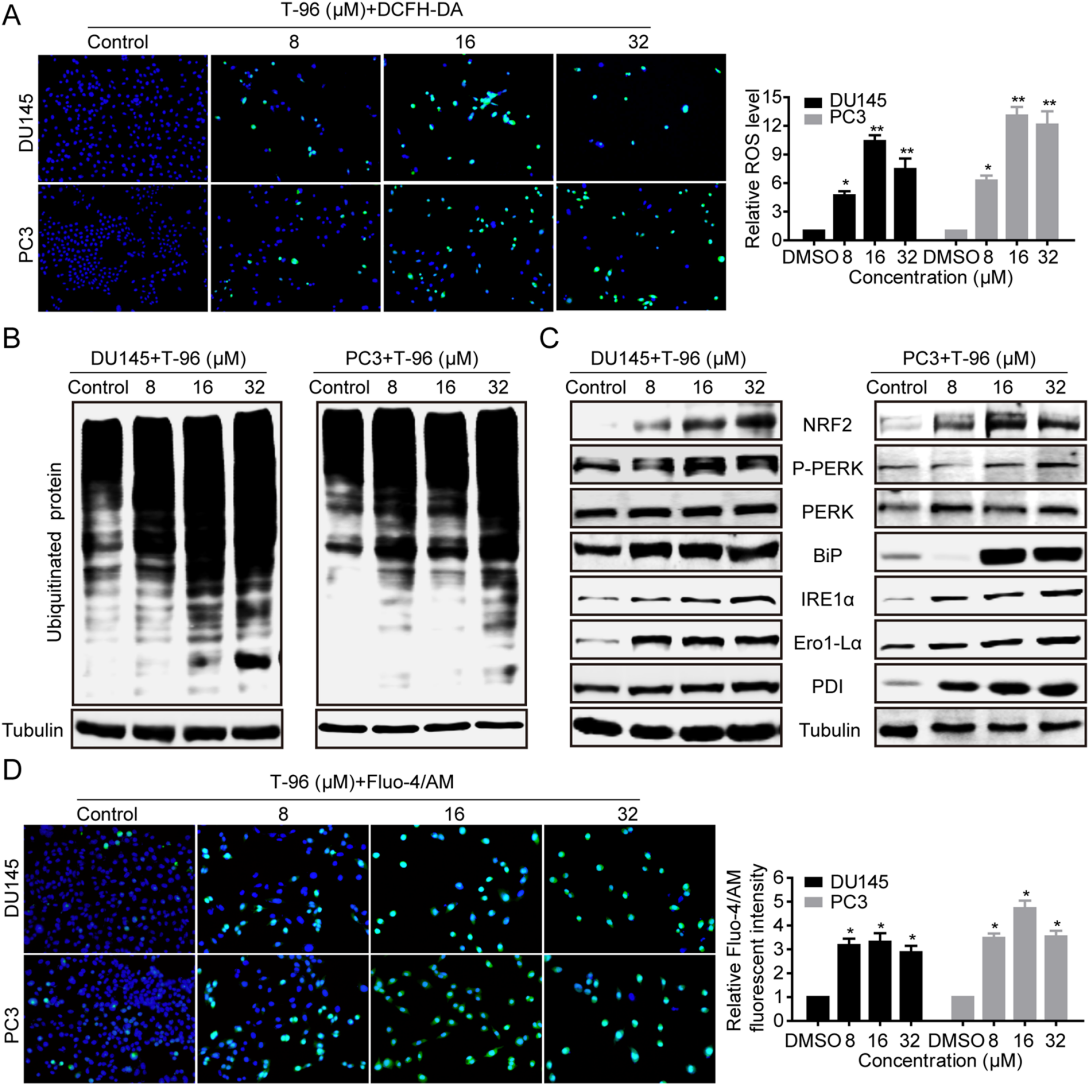
T-96-mediated ROS accumulation induced ER stress. **A** DU145 and PC3 cells were exposed to the indicated concentrations of T-96 for 48 h, and then treated with DCFH-DA for another 30 min. Immunofluorescence analysis was performed to examine the green signal representing ROS generation. Green signal cells were counted and presented in the histogram. Scale bar: 100 μm. **B** T-96 induced accumulation of ubiquitinated proteins. DU145 and PC3 cells were treated with indicated concentrations of T-96 for 48 h, and the ubiquitination levels were then detected using western blot. **C** T-96 activated the UPR pathway in a dose dependent manner. UPR pathway-related proteins were detected using western blot. **D** T-96 enhanced intracellular Ca^2+^ concentration in a dose dependent manner. DU145 and PC3 cells were exposed to the indicated concentrations of T-96 for 24 h, and then cells were incubated with 5 µM Fluo-4/AM for 1 h and detected using fluorescence microscopy. Data are presented as the means ± SD. *P < 0.05 and **P < 0.01 compared with the control group

Previous studies have revealed that ROS is involved in the development of ER stress and the induction of cell death [31–33]. To determine if T-96 induced the generation of ROS, DU145 and PC3 cells were incubated with the specific ROS-detecting fluorescent dye, 2′,7′-dichlorofluorescein diacetate (DCF-DA), and ROS generation was measured using fluorescence microscopy. Different concentrations of T-96 induced significant production of ROS compared to the control group (Fig. 4A). Similarly, T-96 induced the expression of the oxidative stress responsive gene, nuclear factor-like 2 (NRF2), in a dose dependent manner, suggesting the occurrence of excessive accumulation of ROS within CaP cells (Fig. 4C). We further investigated if T-96 promoted ER stress by inducing accumulation of excessive intracellular ROS. Since the accumulation of polyubiquitinated proteins is an important marker of ER stress, we used western blot analysis to evaluate if T-96 affects the levels of total polyubiquitinated proteins in DU145 and PC3 cell lines. As shown in Fig. 4B, T-96 caused a remarkable increase in the levels of polyubiquitinated protein levels in a dose-dependent manner in DU145 and PC3 cells. UPR is an intracellular signaling pathway that is activated when there is accumulation of misfolded and unfolded proteins, and restores ER function by increasing protein folding ability and degrading the accumulated proteins [9, 10, 34]. T-96 increased the phosphorylation of PERK and significantly upregulated the protein levels of several UPR associated proteins, such as BIP, IRE1α, Ero1-L1α and PDI, in a dose-dependent manner (Fig. 4C). ER stress is associated with alteration in Ca^2+^ homeostasis which in turn aggravates the ER stress leading to apoptotic cell death [35]. Therefore, we evaluated whether T-96 disturbs Ca^2+^ homeostasis by staining CaP cells with Furo-4/AM, a Ca^2+^ specific indicator, after treating the cells with different concentrations of T-96. Interestingly, T-96 increased intracellular fluorescence, suggesting that T-96 induced ER stress and the subsequent release of Ca^2+^ from the ER to the cytoplasm (Fig. 4D). In summary, these results demonstrated that T-96 induced ROS generation, which initiated ER stress and the subsequent accumulation of intracellular Ca^2+^.

### T-96 initiated autophagy but blocked autophagic flux to induce apoptosis

**Fig. 5:**
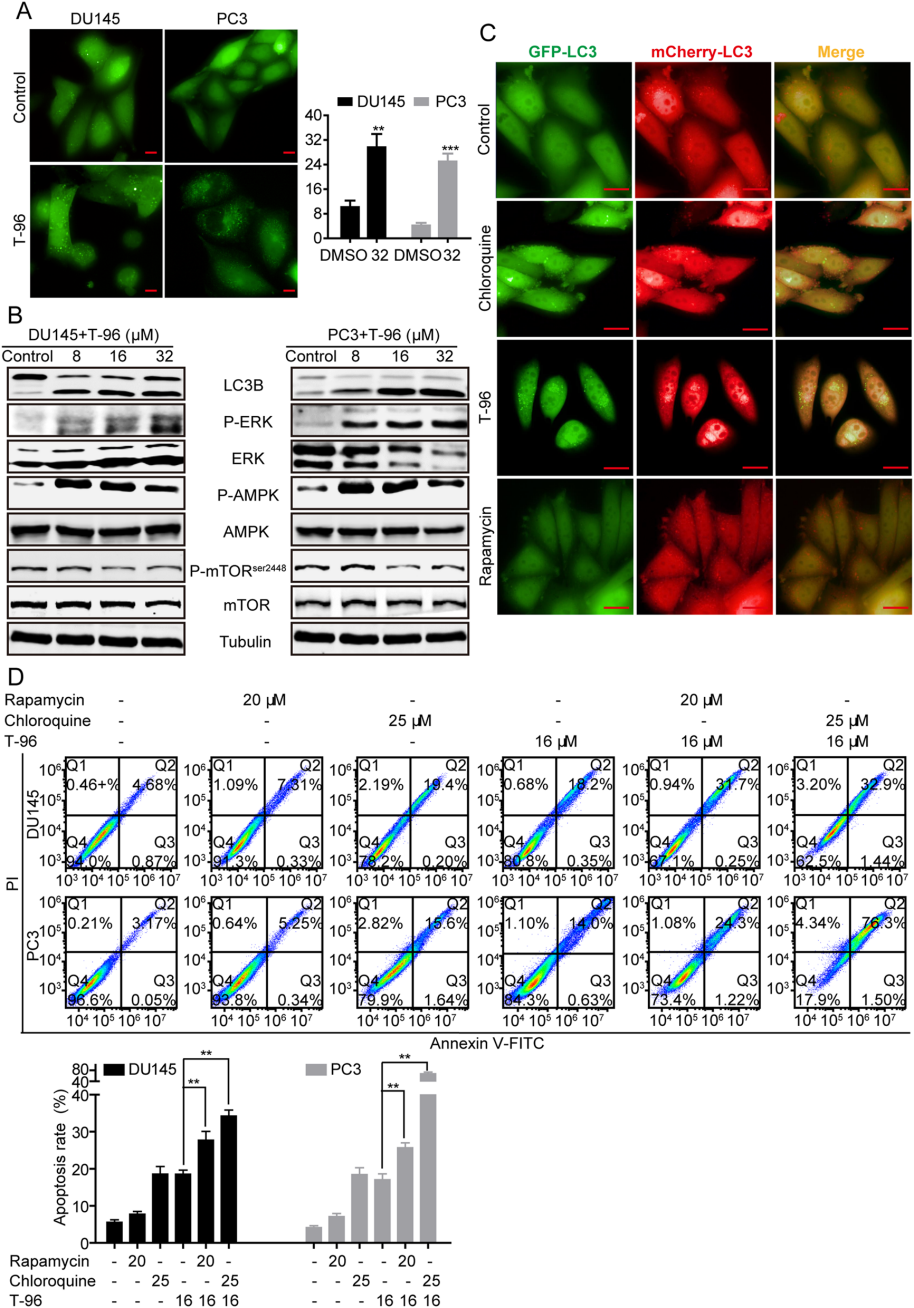
T-96 initiated autophagy but blocked autophagic flux in human CRC cells. **A** The punctate staining pattern of LC3 was detected using high content analysis system-operetta CLS™ in DU145 and PC3 cells after treatment with increasing concentrations of T-96 for 48 h. Scale bar, 10 μm. **B** The expression levels of proteins involved in initiation of autophagy were detected after exposure to T-96 using western blot. β-Tubulin was used as a loading control. **C** T-96 suppressed the autophagic flux in PC3 cells overexpressing mCherry-EGFP-LC3. Both cells were treated with indicated concentrations of T-96 for 24 h and then observed with fluorescent microscopy. Scale bar, 10 μm. Quantitative analysis of the autophagosome accumulation in T-96-treated cells. Yellow dots representing the autophagosomes were counted in three independent experiments. Red fluorescence indicates autophagolysosomes. **D** The suppression of Autophagic flux significantly increased apoptosis. DU145 and PC3 cell lines were treated with rapamycin, chloroquine, T-96, rapamycin in combination with T-96, and chloroquine in combination with T-96. The rate of apoptosis was measured using flow cytometry. Data represent the mean ± SD of three independent experiments. **P < 0.01 and ***P < 0.001 compared with the control group

Several studies have demonstrated that autophagy is activated during ER stress to promote the engulfment of stressed ER by autophagosome [11, 12, 36]. We investigated if T-96 treatment regulates autophagy. The induction of autophagy can be confirmed through the detection of punctate LC3 signals using immunofluorescence, or determining the LC3-II/LC3-I ratio using western blot. First, we used DU145 and PC3 cells transiently transfected with GFP-tagged LC3 to demonstrate the regulatory effect of T-96 on autophagy. From this assay, we observed that T-96 dramatically induced accumulation of GFP-LC3 puncta, which represents the number of autophagic vacuoles, and increased the LC3-II/LC3-I ratio, an indication of increased conversion of LC3-II in a dose-dependent manner in both DU145 and PC3 cells (Fig. 5A, B). The three main pathways involved in the regulation of autophagy are adenosine monophosphate-activated protein kinase (AMPK), mammalian target of rapamycin (mTOR) and mitogen-activated protein kinases (MAPK/ERK) signaling pathways [37, 38]. We next explored whether the T-96-induced initiation of autophagy was effected through the AMPK, mTOR or MAPK/ERK pathways. We found that T-96 significantly increased the phosphorylation levels of AMPK and MAPK/ERK but reduced the phosphorylation levels of mTOR in CaP cells in a dose-dependent manner (Fig. 5B). There is accumulating evidence that suggests that drug-mediated activation of MAPK/ERK can impair autophagy maturation by disrupting the fusion between autophagosome and lysosome [39, 40]. Therefore, we transfected CaP cells with double tagged mCherry-GFP-LC3B reporter to determine if T-96 modulates autophagic flux by regulating the fusion efficiency of autophagosome and lysosome. Yellow fluorescence represented the number of non-acidic autophagosomes, whereas red fluorescence labeled autolysosomes. There was remarkable increase in the number of yellow fluorescent vesicles in DU145 and PC3 cells exposed to T-96 for 12 h compared to the control (Fig. 5C). This implied that there was a defect in the fusion between autophagosome and lysosome which perturbed autophagic flux. It has been previously reported that inhibition of autophagic flux leads to the accumulation of polyubiquinated proteins which subsequently induces ER stress [9, 10, 14, 31–33]. Consistent with these reports, we found that the combination of T-96 and rapamycin, an inhibitor of mTOR, slightly increased apoptosis compared to T-96 treatment alone. In contrast, the combination of T-96 and chloroquine, an autophagic flux inhibitor that decreases autophagosome–lysosome fusion, significantly induced apoptosis in CaP cells implying that inhibition of autophagic flux enhanced T-96-mediated induction of apoptosis (Fig. 5D). Taken together, these results revealed that T-96 initiates autophagy but suppresses autophagic flux, which induces apoptosis in DU145 and PC3 cells.

### T-96 enhanced sensitivity of CaP cells to cisplatin through induction of ER stress and apoptosis

**Fig. 6:**
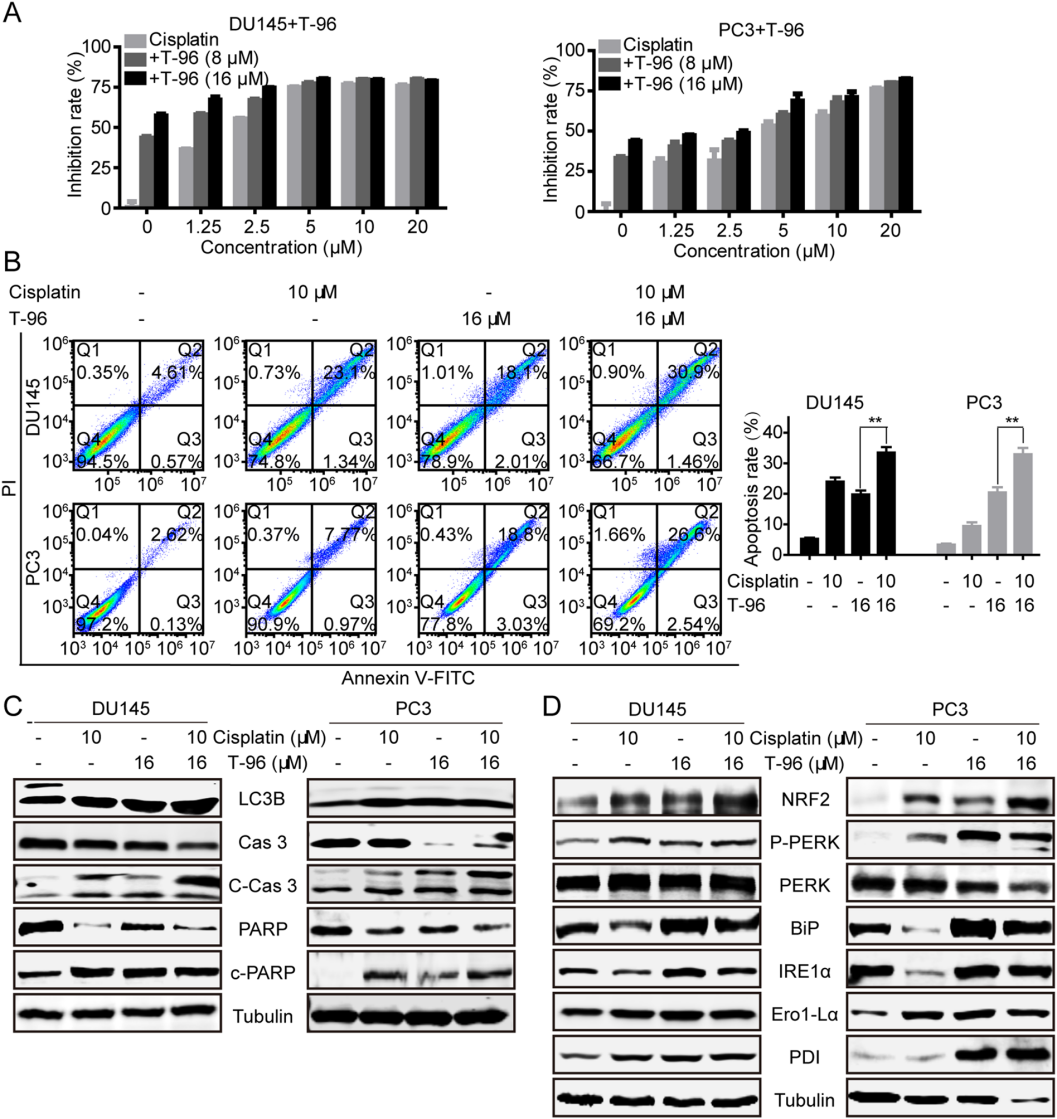
T-96 enhanced sensitivity of human CaP cells to cisplatin. **A** T-96 enhanced the cytotoxic effect of cisplatin against CaP cells. DU145 and PC3 cells were treated with indicated concentrations of T-96 and cisplatin for 48 h. MTT was conducted to assess the effect of T-96 on cisplatin-induced cytotoxicity in CaP cells. **B**, **C** Combined T-96 and cisplatin treatment induced a higher rate of apoptosis than that of monotherapy. DU145 and PC3 cells were treated with T-96, cisplatin, and T-96 combined with cisplatin for 48 h, and then flow cytometry was used to assess the level of apoptosis-induced cell death. The expression levels of indicated proteins were detected using western blot. **D** T-96 and cisplatin co-treatment was more effective in initiating oxidative stress response and UPR. Western blot was used to examine the levels of NRF2 and the UPR associated proteins after treatment with T-96 combined with cisplatin. β-Tubulin was used as a loading control. Data represent the mean ± SD of three independent experiments. **P < 0.01 compared with the control group

Several studies have demonstrated that chemotherapy or radiotherapy-induced autophagy may lead to the development of drug resistance thus protecting cancer cells from apoptosis. On the other hand, the suppression of autophagic flux can improve the sensitivity of cells to chemotherapy as a result of induction of cell death [41–43]. Since our previous assays had demonstrated that T-96 modulates autophagy, we explored the effect of T-96 on the sensitivity of CaP cells to chemotherapy drugs, by treating CaP cells with T-96 alone or in combination with cisplatin. We examined the growth-inhibitory effects of T-96 and cisplatin co-treatment in DU145 and PC3 cells exposed to various concentrations of T-96 (0, 8, and 16 µM) and cisplatin (0, 1.25, 2.5, 5, 10 and 20 µM) alone or in combination for 48 h. Results of the MTT assay showed that co-treatment of DU145 and PC3 cells with T-96 and cisplatin significantly suppressed cell proliferation, whereas monotherapy was associated with low levels of cytotoxicity (Fig. 6A). Next, we evaluated the effect of T-96 and cisplatin, co-treatment on the induction of apoptosis in CaP cells and found that combined treatment caused a higher apoptotic rate than that of monotherapy (Fig. 6B). Consistently, the results of western blot showed that T-96 and cisplatin co-treatment increased the levels of C-Cas3 and PARP compared to treatment with cisplatin alone (Fig. 6C). In addition, T-96 and cisplatin co-treatment up-regulated the expression of NRF2 and the UPR associated proteins, such as p-PERK, BIP, IRE1α, Ero1-L1α and PDI compared to the expression under single-agent treatment (Fig. 6D). This indicated that co-treatment was more effective in initiating oxidative stress response and UPR than monotherapy. Taken together, these results suggest that T-96 improved the chemosensitivity of CaP cells to cisplatin.

## Discussion

Although there have been significant advances in therapeutic strategies against CaP, the emergence of androgen-independence has increased the incidences of unfavourable prognosis and resistance to radio- and chemo-therapy [4]. Therefore, there is urgent need to develop novel agents that can inhibit cell growth and proliferation, overcome multidrug resistance and reduce the recurrence of CaP cells. It has been reported that T-96 has anti-cancer effects against various malignant carcinomas, including melanoma [24], glioblastoma [25], breast cancer [26] and pancreatic cancer [27]. However, its effects on cell growth and possible molecular mechanisms against CaP have not yet been explored. In the current study, we demonstrated that T-96 was a potent inhibitor of CaP by inducing cell cycle arrest at S-phase and activating the extrinsic-apoptosis signalling pathway. Mechanistically, T-96 induced ER stress, initiated autophagy but suppressed autophagic flux and induced apoptosis of CaP cells through the extrinsic pathway. This implies that the ER stress-induced suppression of autophagic flux after treatment with T-96 promoted caspase 8-dependent external apoptosis pathway to inhibit CaP cells. In addition, our findings indicated that T-96 works in synergy with cisplatin to enhance cisplatin-mediated cytotoxicity in CaP cells.

It has been reported that perturbations in ER functions induced by changes in tumor microenvironment or the effects of anti-tumor drugs can trigger ER stress, can lead to the accumulation of unfolded proteins in the ER lumen [44, 45]. ROS overload is the most important inducer of ER stress in the cells [46]. ER stress plays opposing roles in tumour, by either promoting cell survival or inducing cell death. Indeed, it was indicated that low levels of UPR and ER stress act as an accommodative mechanism to promote survival of cancerous cells by inducing multiple antiapoptotic mechanisms. This protects the cells from chemotherapy-mediated cell death, and thus plays a major role in inducing chemotherapy drug resistance [47–49]. On the other hand, excessive levels of ER stress overcome the protective capability of UPR and induce apoptosis of the cancerous cells [49–51]. In line with that, we found that T-96 treatment remarkably induced high levels of ROS, up-regulated the expression of the UPR hallmarks p-PERK, IRE1α, Ero1-L1α and PDI, and significantly increased intracellula Ca^2+^ levels in CaP cells. This suggests that T-96 treatment induced ER stress.

In addition, ER stress is associated with the initiation of autophagy during normal physiological and pathological states as well as during chemotherapy [52–55]. Autophagy is a vital, dynamic and evolutionarily conserved process aimed at elimination of unnecessary or dysfunctional cellular components through degradation in autophagolysosomes. Autophagy has a double-edged sword role in cancerous cells as it can either increase or decrease survival of the cells [56, 57]. Autophagy is mainly induced when serine/threonine kinase UNC-51-like kinase 1 (ULK1) is phosphorylated by either AMPK or mTOR [58]. We found that exposure to T-96 remarkably decreased the levels of LC3I protein and p-mTOR (Ser2448), and dramatically increased the levels of p-AMPK (Thr172) in CaP cells, indicating that T-96 induced autophagy through the AMPK and mTOR pathways. However, some reports have indicated that suppression of mTOR blocks the autophagic flux leading to the accumulation of autophagosomes rather than initiating autophagy [59, 60]. This is consistent with our observation that down-regulation of mTOR phosphorylation was vital for T-96-induced impairment of autophagic flux.

MAPK/ERK activation regulates various cell responses such as proliferation, migration and differentiation [61]. Although high levels of MAPK/ERK phosphorylation have been detected in many malignant tumors, MAPK/ERK activation is not always involved in promotion of cell survival as it also plays a role in processes associated with cell death such as autophagy, apoptosis and senescence [61, 62]. However, the exact effect of MAPK/ERK on activation and maturation of autophagy has not been defined and generates controversial results [38]. Increasing evidence demonstrates that drug treatment prolongs the activation of MAPK/ERK pathway which subsequently blocks the maturation process of autophagy [39, 40]. This is consistent with findings from our study indicating that treatment with T-96 activated autophagy and ERK but impaired autophagic flux. We inferred that T-96-mediated activation of ERK plays an important role in the initiation of autophagy and blockage of autophagic flux. Moreover, activation of MAPK/ERK has been reported to impair autophagy process by promoting the degradation of Fork head Box O1 (FOXO1), a protein associated with maintenance of autophagic flux in cancer cells [63, 64]. It will be interesting to evaluate whether the T-96-induced activation of MPAK/ERK is effected by targeting FOXO1 and determine how ER stress initiates autophagy and impairs autophagic flux in CaP cells.

Recently, growing results have indicated that chemotherapy or radiotherapy-induced autophagy can lead to development of drug resistance whereas the impairment of autophagic flux can improve the sensitivity of cells to chemotherapy by inducing cell death [41–43]. Thus, novel autophagy inhibitors are potential candidates for increasing the efficacy of chemotherapeutic drugs against cancer. In the current study, we found that T-96 enhanced the sensitivity of CaP cells to cisplatin by regulating ER stress-induced autophagy. Therefore, since T-96 exhibits anticancer activity, the combination of T-96 with other chemotherapeutic drugs may be an effective therapeutic strategy for cancer treatment.

## Conclusions

In conclusion, our study demonstrates that T-96 inhibits the survival of CaP cells by inducing cell cycle arrest and apoptosis and works synergistically with cisplastin to inhibit growth of CaP cells. This effect may be due to the induction of prolonged ER stress, which regulates autophagy and activates apoptosis. Our findings suggest that T-96 is a specific regulator of ER stress and autophagy and is a potential candidate for adjuvant therapy for CaP.

## Supplementary Information

**Additional file 1: Figure S1.**The anti-proliferative activity of T-96 onhuman CaP lines DU145 and PC3. (**A**)The chemical structure of T-96. (**B**)The inhibition rates of T-96 on DU145 and PC3 cells. Both DU145 and PC3 cellswere exposed to indicated concentrations of T-96 for 24, 48 and 72 h. MTT wasperformed to measure cell viability and growth. (**C**) IC_50_values of T-96 in DU145 and PC3 cells were calculated. (**D**)IC_50_ valueof T-96 in normal adult prostatic epithelial cell line PNT1A. (**E**) IC_50_ values of cisplatinin DU145 and PC3 cells.Cisplatin was used as a positive drug.

## Data Availability

The datasets and materials obtained and analyzed during the current study were available from the corresponding authors in a reasonable request.

## Abbreviations

AMPK: Adenosine monophosphate-activated protein kinase
BiP: Immunoglobulin heavy chain binding protein
ER: Endoplasmic reticulum
EdU: 5-Ethynyl-2′-deoxyuridine
IRE1α: Inositol-requiring enzyme 1α
mTOR: Mammalian target of rapamycin
MAPK/ERK: Mitogen-activated protein kinases
NRF2: Nuclear factor-like 2
PERK: Double-stranded RNA-dependent protein kinase (PKR)-like endoplasmic reticulum kinase
PDI: Protein-disulfide isomerase
UPR: Unfolded protein response
