# Supplementary figures and images for: Demethylzeylasteral (T-96) initiates extrinsic apoptosis against prostate cancer cells by inducing ROS-mediated ER stress and suppressing autophagic flux
Source: Biol Res. 2021 Sep 6;54:27. doi: 10.1186/s40659-021-00350-6 (PMC8420005; doi:10.1186/s40659-021-00350-6)

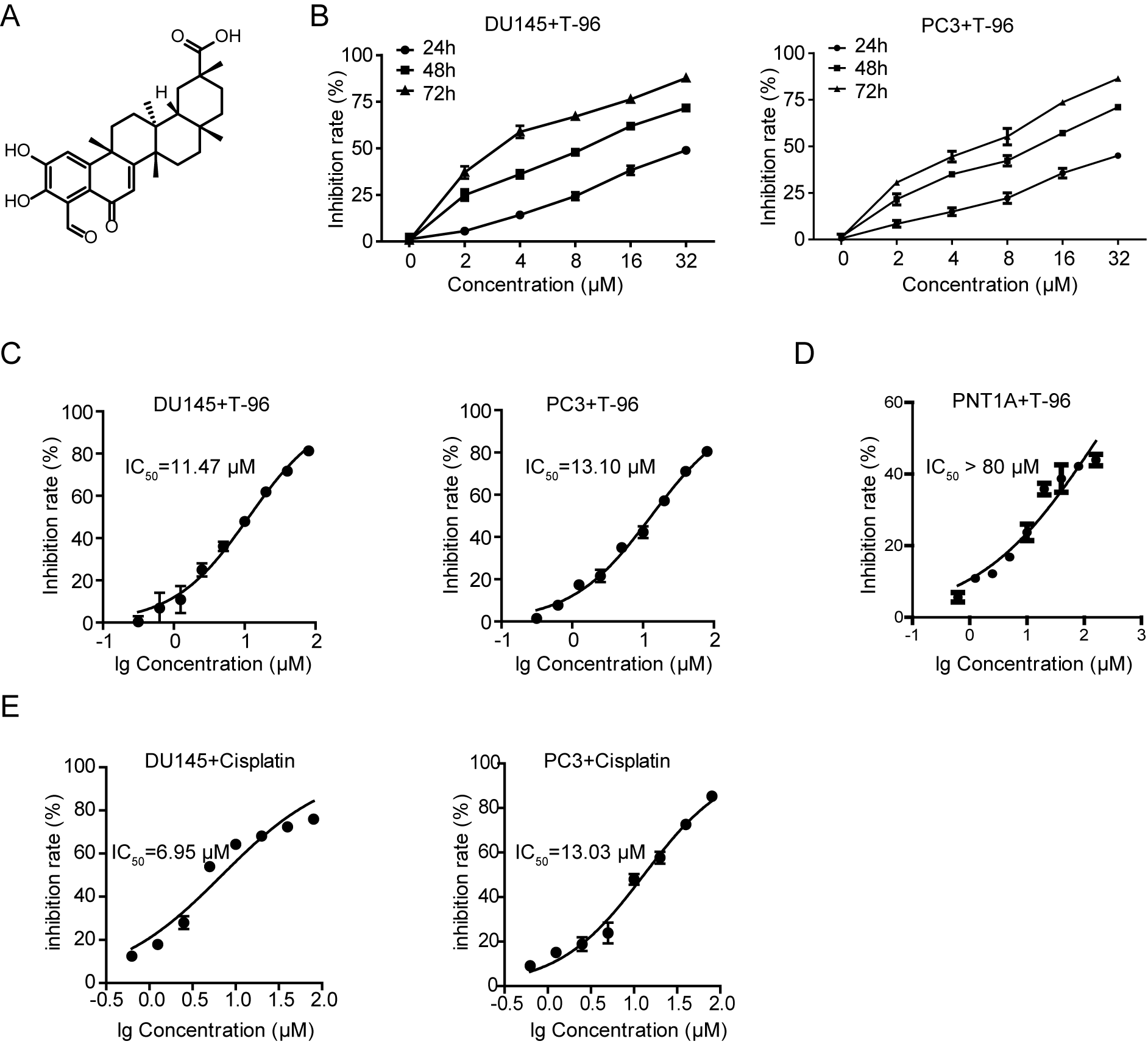

Supplement: Supplementary file 1 — Additional file 1: Figure S1.The anti-proliferative activity of T-96 onhuman CaP lines DU145 and PC3. (A)The chemical structure of T-96. (B)The inhibition rates of T-96 on DU145 and PC3 cells. Both DU145 and PC3 cellswere exposed to indicated concentrations of T-96 for 24, 48 and 72 h. MTT wasperformed to measure cell viability and growth. (C) IC50values of T-96 in DU145 and PC3 cells were calculated. (D)IC50 valueof T-96 in normal adult prostatic epithelial cell line PNT1A. (E) IC50 values of cisplatinin DU145 and PC3 cells.Cisplatin was used as a positive drug. [file 40659_2021_350_MOESM1_ESM.tif]
